# Molecular spin-probe sensing of H-mediated changes in Co nanomagnets

**DOI:** 10.1126/sciadv.ads1456

**Published:** 2025-02-12

**Authors:** Alex Fétida, Olivier Bengone, Christine Goyhenex, Fabrice Scheurer, Roberto Robles, Nicolás Lorente, Laurent Limot

**Affiliations:** ^1^ Université de Strasbourg, CNRS, IPCMS, UMR 7504, F-67000 Strasbourg, France.; ^2^ Centro de Física de Materiales CFM/MPC (CSIC-UPV/EHU), Paseo Manuel de Lardizabal 5, 20018 Donostia-San Sebastián, Spain.; ^3^ Donostia International Physics Center (DIPC), 20018 Donostia-San Sebastián, Spain.

## Abstract

The influence of hydrogen on magnetization is of substantial interest to spintronics. Understanding and controlling this phenomenon at the atomic scale, in particular in nanoscale systems, is crucial. In this study, we used scanning tunneling microscopy (STM) combined with a nickelocene molecule to sense the spin of a hydrogen-loaded nanoscale Co island grown on Cu(111). Magnetic exchange maps obtained from the molecular tip revealed the presence of a hydrogen superstructure and a 90° rotation of the magnetization compared to the pristine island. Ab initio calculations corroborate these observations, indicating that hydrogen hybridization with Co atoms on the island surface drives the spin reorientation of the island. This reorientation is further reinforced by hydrogen penetration into the island that locates at the Co/Cu interface. However, the subsurface sensitivity of the magnetic exchange maps indicates that this effect is limited. Our study provides valuable microscopic insights into the chemical control of magnetism at the nanoscale.

## Teaser

A spin-sensitive molecular probe enables high-resolution imaging of nanoscale magnetization in hydrogen-based systems.

## INTRODUCTION

Magnetic anisotropy determines the orientation of spins in metallic thin films and multilayers, thereby constituting a critical aspect of spintronic device functionality. Among various approaches for controlling magnetic anisotropy, the incorporation of mobile ionic species in thin films through voltage-gated transport stands out as promising. Hydrogen, in particular, has proven superior to other ionic species, offering nondestructive and rapid toggling of magnetic anisotropy of multilayered heterostructures (*1*–*3*). The rationale for hydrogen loading is built upon the observation that even subtle interactions, such as charge transfer between hydrogen and a metal atom, can trigger changes in the magnetic anisotropy and effective magnetic moment of the atom (*4*–*9*), while also affecting the exchange coupling among atoms (*10*, *11*). The magnetization orientation in ultrathin films can be modified by the adsorption and penetration of hydrogen (*10*–*14*). This effect is observed not only in collinear magnetic films but also in noncollinear ones (*15*, *16*), where hydrogen has the ability to stabilize skyrmion states (*17*, *18*). The position and concentration of hydrogen in and on the host metal are important factors in this process (*17*, *19*). While recent advancements have enabled microscopic-level imaging of hydrogen (*20*), these measurements have not yet been associated with magnetism.

Single hydrogen molecules are commonly studied using scanning tunneling microscopy (STM). Hydrogen molecules assemble into coverage-dependent lattices on metal surfaces (*21*) and have a characteristic vibrational structure facilitating their identification (*21*, *22*). They can also be manipulated by the STM tip (*23*–*25*), adding chemical functionality to the microscope (*26*). Dissociative adsorption of hydrogen molecules has also been reported on magnetic surfaces, where hydrogen atoms then form superlattices (*27*, *28*). However, conducting spin-sensitive STM measurements on hydrogen-exposed magnets remains a challenging endeavor (*17*, *28*), as it necessitates a magnetic tip, which, similar to the surface, is susceptible to hydrogen contamination (*29*).

## RESULTS

**Fig. 1.**
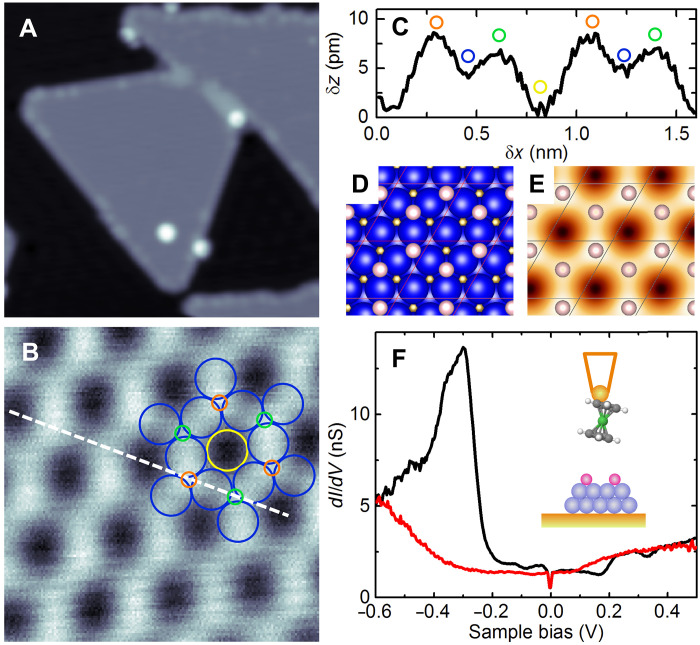
Topography and tunneling spectroscopy of hydrogen superstructure on a Co island. (**A**) Constant-current image of H-covered Co bilayer islands on Cu(111) (image size, 20 × 20 nm^2^; sample bias, 50 mV; and tunnel current, 50 pA). The bright spots are Nc molecules. (**B**) Close-up view of the island (1.7 × 1.7 nm^2^; sample bias, 50 mV; and tunnel current, 50 pA) with 2H-(2 × 2) superstructure [Co(I), yellow; Co(II), blue; H(I), green; and H(II), orange]. The Co network corresponds to that of the pristine island before hydrogen exposure. (**C**) Height profile acquired along the dashed line indicated in (B). Colored circles indicate the position of the H and Co atoms. (**D**) Model structure of 2H-(2 × 2) (Co, blue; and H, white) and (**E**) corresponding DFT-computed constant-current topography (simulation done at 100 mV). Circles represent H atoms. (**F**) Typical *dI*/*dV* spectra acquired on H-contaminated (solid red line) and pristine islands (solid black line). Feedback loop opened at 1.2 nA and 0.5 V. Inset: sketch of the tunnel junction.

The need for atomic-scale imaging of hydrogen and its impact on magnetism calls for innovative scanning probe methods that are resilient to hydrogen contamination. Here, we propose using a molecular spin-sensitive tip. In particular, we passivated the apex of a copper-coated W tip with a magnetic nickelocene molecule [Ni(C_5_H_5_)_2_, denoted as Nc, see Fig. 1F], and focused on a model system comprising a nanoscale Co magnet that was exposed to hydrogen. Owing to the direct exchange coupling between the Nc-terminated tip and the Co surface of the magnet, we used the inelastic tunnel current to monitor the orientation of the surface magnetization with atomic-scale sensitivity (*30*, *31*). Magnetic exchange maps obtained with the molecular tip reveal that hydrogen is predominantly located on the magnet’s surface, forming a hydrogen superstructure, while the subsurface sensitivity of the magnetic probe tip indicates weaker hydrogen penetration into the magnet. This results in a 90° rotation of the magnetization compared to the hydrogen-free state. These observations, corroborated by ab initio calculations, represent an advancement in our comprehension of how hydrogen alters the magnetism of a nanoscale system at the atomic level.

The sample consists of Co islands grown on Cu(111), which are two atomic layers high and exhibit a triangular-like structure (Fig. 1A) (*32*–*34*). The apparent height is 325 ± 10 pm, and typical lateral sizes range from 10 to 30 nm. After island growth, Nc is deposited onto the sample, which is then exposed to hydrogen (see Materials and Methods for details). To assess H-adsorption onto Co islands, a Nc tip is prepared by transferring a single Nc from the surface to a mono-atomically sharp tip. Imaging can then be conducted via two operating modes. The first mode involves tunneling with parameters 50 mV and 50 pA, for which the exchange interaction with the Co surface is absent (see fig. S1 and discussion in Supplementary Text). In this mode, which we define as a high-bias mode, the Nc tip functions as a standard STM tip. The second mode, which we define as a low-bias mode, involves tunneling with parameters 1 mV and 100 pA, where the Nc tip is 50 pm away from the magnetic surface for the exchange interaction to be active. This mode carries magnetic information. Switching between high and low bias allows us to visualize the electronic and magnetic properties, respectively.

### Hydrogen superstructure on a Co bilayer island

To determine the hydrogen coverage on a nanoscale island, we used the Nc tip in the high-bias mode. Figure 1A shows a typical image of an island after hydrogen exposure (see Materials and Methods), which appears similar to a pristine island. To visualize the presence of hydrogen atoms, it is necessary to zoom in on the island. A close-up view of a Co island is presented in Fig. 1B, along with an overlaid simulation of the Co network of the pristine island. Hydrogen atoms are positioned on the hollow sites of the Co surface and alternate between two distinct hollow sites, resulting in a 2 pm difference in their apparent height (Fig. 1C). The hydrogen coverage is 0.5 monolayers (ML), suggesting a hydrogen arrangement in a 2H-(2 × 2) superstructure (Fig. 1D). The unit cell of the superstructure contains two Co atoms: one at the center of a hexagon without neighboring H atoms [noted Co(I), yellow in Fig. 1B], and a second, which has two neighboring H atoms [noted Co(II), blue]. The apparent height of Co(II) is 4 pm greater than that of Co(I). To corroborate these observations, density functional theory (DFT) calculations were carried out, yielding good agreement with the experimental images (Fig. 1E). The weaker corrugation in the computed image corresponds to hydrogen in a hexagonal close-packed (hcp) site [noted H(I), green circle in Fig. 1B], while the stronger corrugation corresponds to hydrogen in a face-centered cubic (fcc) site [noted H(II), orange circle].

This assignment, however, needs to be validated also by tunneling spectroscopy. The 1H-(2 × 2) superstructure, where one H atom is present in a (2 × 2) Co unit cell corresponding to a 0.25 ML coverage, is indistinguishable from its 2H counterpart in the computed images (see fig. S2). Figure 1F presents a typical *dI*/*dV* spectrum of a pristine Co island, revealing a prominent peak at −0.33 eV, arising from minority $d_{z^{2}}$ states hybridized with *s-p* states (*31*). The sharp feature at zero-bias is due to the inelastic tunnel current of the Nc tip. After H exposure, the peak shifts below −0.6 eV. This behavior is consistent with the computed local density of states (LDOS) for both Co(I) and Co(II) in the 2H-(2 × 2) superstructure (see fig. S2). It contrasts with the LDOS of a 1H-(2 × 2) superstructure, which maintains instead a spin-polarized *d*-structure comparable to the pristine surface (see fig. S2). Hence, the islands in this study exhibit a 2H-(2 × 2) superstructure after hydrogen exposure.

### Magnetization in the presence of hydrogen

**Fig. 2.**
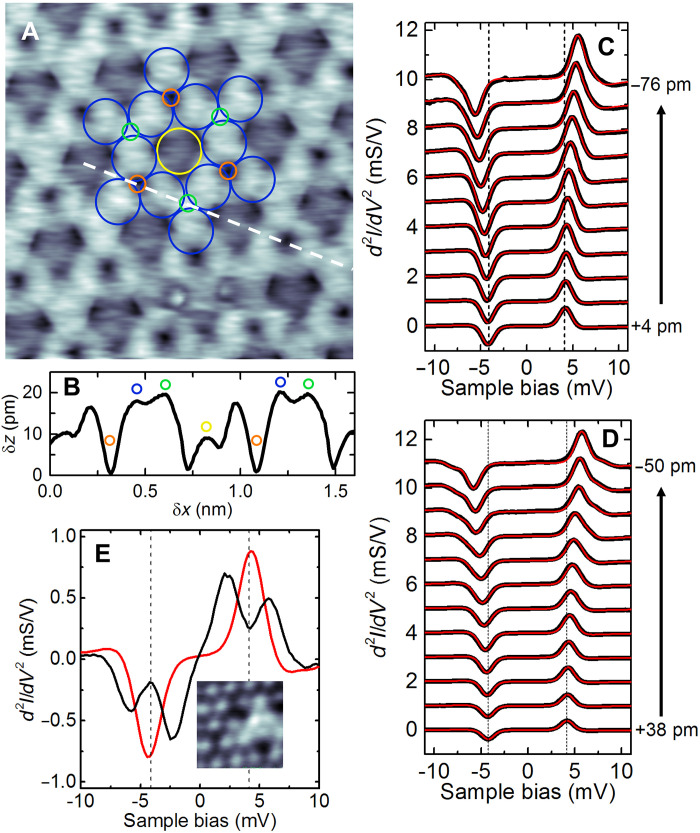
Probing a Co island with a nickelocene tip. (**A**) Low-bias constant-current image of an H-covered Co bilayer island (1.7 × 1.7 nm^2^, 1 mV, 100 pA) with simulated 2H-(2 × 2) superstructure [Co(I), yellow; Co(II), blue; H(I), green; and H(II), orange]. (**B**) Height profile acquired along the dashed line indicated in (A). Colored circles indicate the position of the H and Co atoms. (**C**) and (**D**) present the $d^{2}I/{\mathrm{dV}}^{2}$ spectra above Co(I) and Co(II) atoms for different tip-Co distances. Feedback loop opened above Co(I). Red lines: Simulations based on a dynamical scattering model (*35*). (**E**) $d^{2}I/{\mathrm{dV}}^{2}$ spectra acquired before and after hydrogen removal (solid red line, before; and solid black line, after). The dashed lines indicate the nonshifted peak (dip) position at large distance. Inset: Image acquired after H-removal (1 × 1 nm^2^, 1 mV, 100 pA). Hydrogen removal is achieved through several 1-Vpulses applied for a few seconds at a tunneling current of 10 μA. It specifically targets the island beneath the tip, without perturbing neighboring islands. After the procedure, the Nc tip is lost and must be re-prepared.

To investigate the magnetism of the *2*H-(2 × 2) superstructure, the surface is imaged by using the low-bias mode of the Nc tip. The low-bias image of Fig. 2A differs from the one previously recorded at higher bias. The Co(I) and Co(II) atoms show a stronger contrast due to their distinct corrugation (Fig. 2B), with a measured difference of 8 pm between them. The two hydrogen atoms are also visible, but their contrast is reversed compared to the high-bias image, H(I) now exhibiting a height 20 pm larger than H(II). To determine the orientation of the island magnetization, we recorded a series of $d^{2}I/{\mathrm{dV}}^{2}$ spectra by approaching the tip above each Co atom. The zero tip displacement (δz = 0) is set to a conductance of *G* = 0.01 *G*_0_ above a Co(I) atom, where *G*_0_ = 2*e*^2^/*h* is the quantum of conductance. Conductance versus tip-displacement traces are presented in fig. S3. As shown in Fig. 2C for Co(I) and in Fig. 2D for Co(II), at a tip displacement of δz = 0, the spectra exhibit a dip and a peak at biases of −4.0 and +4.0 mV, respectively. These peaks and dips reflect inelastic scattering events, in which tunneling electrons exchange momentum and energy with the spin states of Nc (*36*). As the tip approaches the surface, the peak (dip) for Co(I) and Co(II) shifts upward (downward) in energy, indicating exchange coupling between the Nc tip and Co. This is also visible in the two-dimensional (2D) intensity plots acquired above both cobalt atoms (see fig. S4). The evolution of the spectra can be rationalized via the following spin Hamiltonian (*31*)

$$\hat{H}={\mathrm{DS}}_{z}^{2}-g\mu_{B}{\hat{B}}_{\text{ex}}\cdot \hat{S}$$ (1)

where ${\hat{B}}_{\text{ex}}$ represents the exchange field produced by the sample atoms on the spin $\hat{S}$ of Nc, μ_*B*_ denotes the Bohr magneton, *g* = 1.89 (*37*), and *D* is the easy-plane magnetic anisotropy of Nc. The spectra are reproduced using a dynamical scattering model with an in-plane exchange field (solid red lines in Fig. 2, C and D) (*35*), with *D* = 4.0 ± 0.1 meV, which is usual for Nc tips (*38*). Peaks and dips at opposite voltage polarities exhibit different amplitudes (refer to Fig. 2E, solid red line). This disparity arises from the selection rule for spin excitation and their intensity ratio provides insight into the magnitude and sign of the spin polarization of the tunnel junction (*39*). The spin polarization (*P*) found from our simulated spectra is constant with tip displacement with *P* = −0.12 for both Co(I) and Co(II) (see discussion and fig. S5). The spectra differ from those of the pristine island. This becomes evident upon removing hydrogen by locally heating the island with the STM tip (*27*). Following hydrogen removal, the Co network is restored (Fig. 2E), and Co atoms display a characteristic spin-split $d^{2}I/{\mathrm{dV}}^{2}$ spectrum, indicating out-of-plane magnetization and nearly zero spin polarization as in a pristine Co bilayer island (*30*, *31*). The 2H-(2 × 2) superstructure can therefore be associated to a rotation of the island magnetization, from out of plane to in plane.

**Fig. 3.**
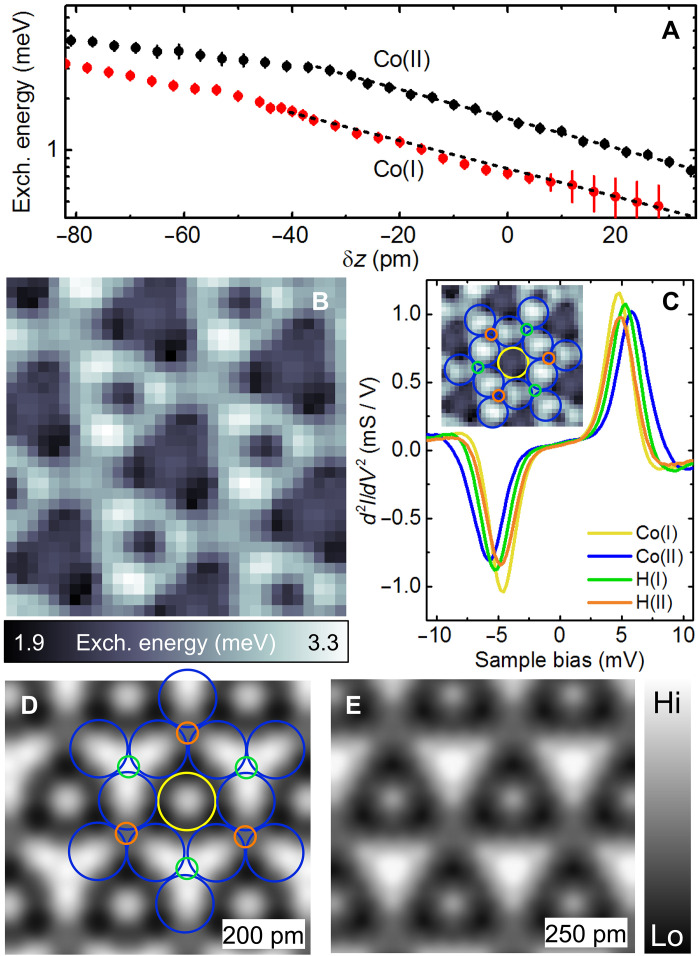
Exchange and computed spin-density maps. (**A**) Exchange energy versus δz extracted from the $d^{2}I/{\mathrm{dV}}^{2}$ spectra of Co(I) and Co(II). The dashed lines represent the exponential fit described in the text. (**B**) Spatial map of the exchange energy acquired at a fixed distance above a Co island (δz = −40 pm). Lateral (vertical) tip drift between each spectrum acquisition was kept below 10 pm (5 pm) by dynamically tracking a local maximum. (**C**) $d^{2}I/{\mathrm{dV}}^{2}$ spectra above Co(I), Co(II), H(I), and H(II) [Co(I), yellow; Co(II), blue; H(I), green; and H(II), orange]. Computed spin density map at a fixed distance of (**D**) 200 pm and (**E**) 250 pm above H(I).

Figure 3A presents the distance-dependent behavior of the exchange energy derived from the spectra above Co(I) and Co(II). For δz > −40 pm, the exchange energy exhibits an exponential variation exp(−δz/λ) with a decay length of λ = 46 ± 10 pm for both atoms, similar to a Co atom of the pristine island (*31*). Notably, the exchange energy above Co(II) is nearly twice as strong as the exchange energy measured above Co(I). When the tip is approached further (δz < −40 pm), the exchange energy becomes less sensitive to tip displacement, showing a smaller decay length; some variation in decay length was observed depending on the tip. We assign this behavior to an Nc-hydrogen repulsion that is more pronounced on Co(II) than on Co(I) (see discussion related to fig. S3 in the Supplementary Text). The exchange coupling also varies across the surface. To visualize these variations, we acquire a voxel image consisting of a dataset in the form of $d^{2}I/{\mathrm{dV}}^{2}\left(x,y,V\right)$, while maintaining a fixed tip-sample distance. We then determine the exchange energy at each lateral tip position by fitting the spectra. Figure 3B shows the resulting magnetic exchange map, while Fig. 3C displays $d^{2}I/{\mathrm{dV}}^{2}$ spectra at the Co and H atoms. The exchange map is similar to the low-bias image of Fig. 2A since the corrugation in both images is governed by the shift of the inelastic threshold energy. If the low-bias image allows quickly assessing the lateral dependence of the exchange coupling, it is done at the expense of a quantitative estimate of the exchange energy. The exchange map reveals a corrugation of the exchange energy over a 1.5 meV range, with clear differences among Co and H atoms. However, these differences are not reflected in their magnetic moments. The DFT-computed magnetic moments are 1.9 μ_*B*_ and 1.6 μ_*B*_ for Co(I) and Co(II), respectively, while the hydrogen atoms exhibit weak magnetism, with H(I) at −0.02 μ_*B*_ and H(II) at −0.01 μ_*B*_.

Consistent with findings on pristine Co surfaces (*31*), the magnetic exchange map is reproduced by the DFT-computed spin density. Figure 3 (D and E) presents the spin density computations at two distinct distances from the surface, based on a 2H-(2 × 2) superstructure on a Co bilayer. This agreement extends to H-covered Co islands of varying Co island thicknesses (see fig. S6). The maps indicate that Co(II) exhibits a stronger spin density compared to Co(I) at all distances from the surface, aligning with the experimentally observed stronger exchange energy. The observed differences in the Co atoms, also visible in their computed LDOS (see fig. S2), are attributed to varying hybridization with the hydrogen atoms. In addition, the spin density of H(II) is stronger than that of H(I), confirming the contrast reversal seen experimentally for these hydrogen atoms compared to the high-bias images.

### Computed magnetic anisotropy energy

To gain deeper insight into our findings, we have performed a computational study of hydrogen adsorption and its impact on the island’s magnetization orientation. The key quantity to calculate is the magnetic anisotropy energy (MAE), which is the sum of two contributions. The first contribution, known as the magnetocrystalline anisotropy energy (MCA), stems from the spin-orbit coupling and dictates the preferred alignment direction of magnetic moments within the material arising from the crystalline structure and symmetry. The second component, termed shape anisotropy, is driven by magnetostatic dipole-dipole interactions and manifests as a tendency for the magnetic moments to align in a specific direction dictated by the object’s shape. Here, a negative MAE corresponds to out-of-plane magnetization (denoted as ⊥), while a positive value indicates in-plane magnetization (denoted as ∥). Calculation details are given in Materials and Methods. To keep the calculations within practical time limits, we use the approximation of infinite bilayers of Co on a Cu(111) substrate. This approach is reasonable given that the triangular islands seen in experiments are larger than 10 nm on each side. The shape anisotropy in a triangle is then almost the same than the one of an infinite film of the same height.

**Fig. 4.**
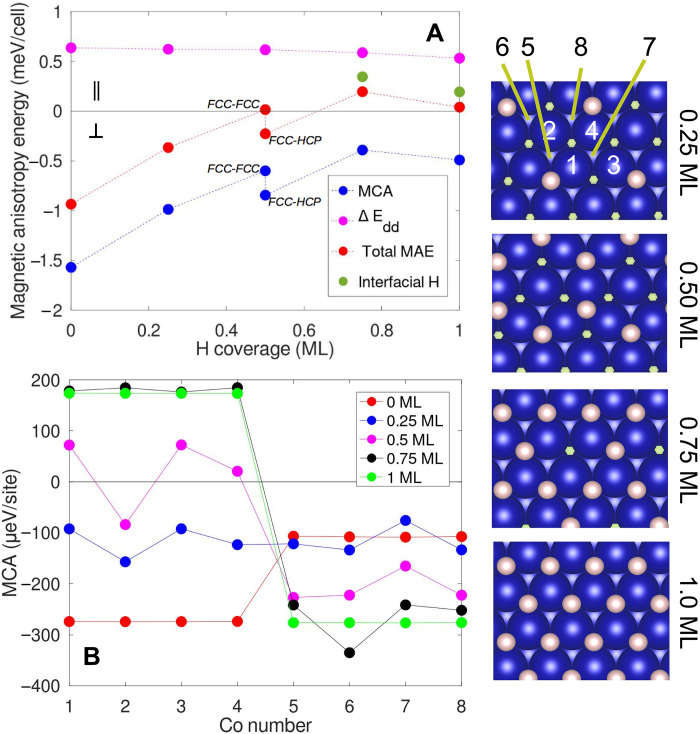
Computed MAE for a Co island and local site analysis. (**A**) MAE versus surface hydrogen coverage (ML). Blue, MCA energy; magenta: shape anisotropy, Δ*E*_*dd*_; red: total MAE. The two additional values represented by the green circles correspond to the MAE of the configurations with 0.5 ML of H at the surface plus 0.25 and 0.50 ML of H at the Co/Cu interface. These values correspond, respectively, to coverages of 0.75 ML and 1 ML (see table S1 for details). For convenience, the energy values are given in meV/cell where the considered Co 2 × 2 cell contains 8 Co atoms. (**B**) Local site analysis of the MCA. The abscissa indicate the number of the Co atom in the cell (right panels): 1 to 4 for the surface layer and 5 to 8 for the first Co layer.

In the first step of this theoretical study, we consider only the hydrogen adsorption on top of the Co bilayer. We vary the hydrogen coverage from 0 to 1 ML and calculate the MAE for each coverage. Since these calculations are conducted using a 2 × 2 − (111) cell configuration, these coverages correspond to the addition of 0 to 4 hydrogen atoms per unit cell on the (111) surface. The H atoms are positioned in hollow fcc sites. For the 0.5 ML coverage, we also consider a superstructure alternating fcc and hcp positions as it is more stable, in agreement with STM experiments. The results are presented in Fig. 4A. For pristine Co (0 ML of H), the MCA is negative and exceeds the shape anisotropy, resulting in an out-of-plane magnetization (MAE < 0). The main effect of adding hydrogen is to bring the MAE close to zero. This is driven by the MCA changing from −1.5 to −0.5 meV per cell, while the shape anisotropy remains constant at 0.6 meV per cell. At a hydrogen coverage of 0.5 ML, the two contributions compensate, resulting in an MAE that is nearly zero. Above 0.5 ML, the further growth of the MCA is slower but sufficient to favor in-plane magnetization. Thus, the MCA evolution with hydrogen coverage is the driving force behind the rotation of the magnetization toward the in-plane configuration.

A deeper understanding of the physical origin can be gained through a Co site–resolved analysis, which involves plotting the MCA for each Co site (Fig. 4B). As the H coverage on the surface increases, the MCA increases at the Co sites on the surface (numbered 1 to 4 in Fig. 4). The MCA becomes large and positive above 0.5 ML, favoring an in-plane orientation of the magnetization. In contrast, the Co sites at the interface with copper (noted 5 to 8 in Fig. 4), which have no hydrogen atoms as nearest neighbors, exhibit opposite behavior when the H coverage is >0.5 ML, thus favoring an out-of-plane magnetization. The different MCA for surface and first-layer Co atoms is attributed to their hybridization with H atoms that changes the relative importance of different *d* orbitals of Co (see fig. S7 and the related discussion in the Supplementary Text), as also evidenced in Pd/Co/Pd thin films (*19*).

### Determining hydrogen loading in the experiment

The above considerations regarding the MCA per Co site lead us to consider the possibility of H insertion as an enhancing factor of the island magnetization re-orientation. We start from a coverage of 0.5 ML where the hydrogen is present only on the surface in a 2H-(2 × 2) superstructure, and progressively insert H atoms in octahedral sites within the island. We find several stable configurations with close energies but with different magnetic anisotropies (see table S1). Loading the Co unit cell with one additional H atom in between the two Co layers (corresponding to a coverage of 0.75 ML) leads to a negative MAE, in disagreement with experiments. Conversely, if the additional H atom is incorporated at the Co/Cu interface, the average MAE is positive, and is further reinforced when inserting 2 H atoms (corresponding to a coverage of 1 ML). The two positive values of MAE have been reported on Fig. 4A as green circles (see table S1). Increasing the concentration above 1 ML involves inserting H at octahedral sites in both Co/Co and Co/Cu interfaces, leading to a limited number of configurations with in-plane magnetization. For concentrations >1.5 ML, the MCA energy becomes positive for all possible configurations. The observed magnetization rotation can thus be attributed to minimal hydrogen coverage of approximately 0.5 ML on the surface of the Co island.

**Fig. 5.**
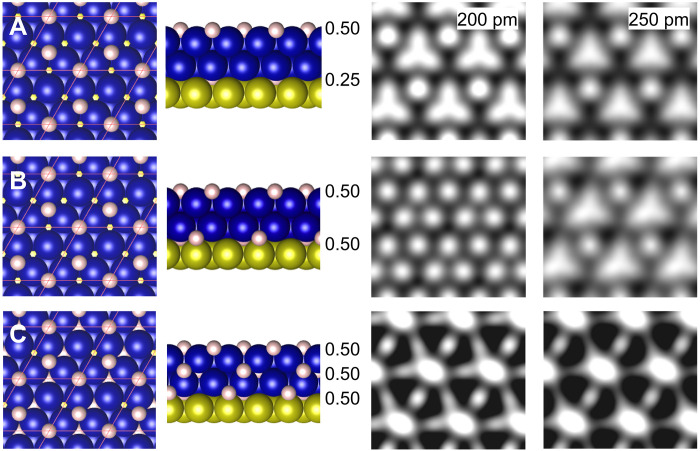
Computed spin-density maps with increasing H coverage. Top and side view of hydrogen loading in a Co bilayer having a 2H-(2 × 2) superstructure on its surface. Two spin density maps are presented, that are computed at two fixed distances above an H atom (left, 200; right, 250 pm). In (**A**) 0.25 ML of hydrogen was added to the Co/Cu interface, (**B**) 0.50 ML of hydrogen was added to the Co/Cu interface, and (**C**) 0.50 ML of hydrogen was added to both the first Co layer and the Co/Cu interface. The H-coverage of (A) and (B) correspond to the coverages plotted as green circles in Fig. 4A.

To establish an upper limit for hydrogen coverage and identify possible H insertion sites in the Co bilayer, we use the previously identified configurations with in plane magnetization, calculate the corresponding spin density maps, and compare them with experimental exchange images. The experimental images reveal hydrogen-induced modifications in both the top and bottom layers of Co atoms (see fig. S8 and the related discussion in Supplementary Text) (*30*). Figure 5A shows computed spin density maps with an extra 0.25 ML of hydrogen at the octahedral sites of the Co/Cu interface. There is no notable difference compared to the experimental map (Fig. 3B). However, increasing the hydrogen to 0.50 ML at the Co/Cu interface (Fig. 5B) and further hydrogen loading (Fig. 5C) result in spin density patterns that diverge from the experimental observations. On the basis of these findings, if hydrogen is present in the island, the total coverage must be between 0.5 ML and 0.75 ML, with 0.5 ML located on the surface.

## DISCUSSION

In summary, our study demonstrates that exposing a nanoscale magnet to sufficient hydrogen can induce a 90° rotation of its magnetization. Although our DFT computations are conducted on infinite Co layers, they successfully reproduce the interplay between hydrogen loading and magnetism observed in finite-size Co magnets, typically few tens of nanometers wide in our measurements. This rotation is primarily driven by hydrogen adsorption on the magnet’s surface and is further reinforced by the presence of hydrogen at the Co/Cu interface. While hydrogen adsorption also occurs at step edges in the experiment, our findings suggest that this effect can be neglected to first order. Notably, high-resolution hydrogen imaging with a spin-sensitive molecular probe tip holds substantial promise for achieving precise control over magnetization at the nanoscale, advancing both our understanding and the practical integration of hydrogen into magnetic systems. This approach could also be extended to a wider array of materials containing elements like oxygen, nitrogen, and carbon, which can similarly influence magnetic anisotropy.

## MATERIALS AND METHODS

### Experimental details

We used a customized Omicron ultrahigh vacuum scanning tunneling microscope with a pressure maintained below 10^−10^ mbar. Inelastic tunneling spectroscopy was performed at 2.4 K, while all other measurements were carried out at 4.4 K. The Cu(111) substrate was cleaned through multiple cycles of Ar^+^ sputtering and annealing at a temperature of 520°C. The W tip used in this investigation was first cleaned by Ar^+^ sputtering. Cobalt islands were grown on Cu(111) by evaporating Co onto the surface at room temperature. Cobalt was evaporated from a rod that had undergone thorough outgassing, with an evaporation rate of 0.3 ML min^−1^. After a deposition of 1 ML, the cobalt sample was transferred to the precooled microscope. Nickelocene was then deposited by exposing the sample, maintained below 100 K, to a molecular flux of 2.5 × 10^−2^ ML min^–1^ for a few seconds.

For the Nc tip preparation, we first indented the tungsten tip into the copper surface, resulting in the creation of a copper-covered, mono-atomically sharp tip apex. Subsequently, we positioned the tip above an Nc molecule adsorbed on either a cobalt or copper step edge. To attach Nc to the tip, the tunneling parameters were set at −1 mV and 50 pA, and the tip was carefully approached toward the molecule by a minimum of 200 pm. Notably, molecular attachment was also possible with Nc molecules adsorbed on the Co islands, using tunneling parameters of 50 mV and 50 pA, and approaching the tip by 350 pm. Details concerning Nc tip characterization can be found elsewhere (*31*). The dI/dV spectra were recorded using a lock-in amplifier operating at a frequency of 6.2 kHz and a modulation of *5* mV rms. The $d^{2}I/{\mathrm{dV}}^{2}$ were recorded using a lower modulation of 500 μV rms.

Following the deposition of nickelocene, we initiated the hydrogenation of the sample. Hydrogen molecules constitute the predominant residual gas in the UHV chamber. In our UHV environment, where the pressure is <5 × 10^−11^ mbar, hydrogen contaminates the Co islands over time. Achieving the desired hydrogen superstructure on the islands requires however an “efficient” hydrogenation. This consists in increasing the temperature of the cryostat to more than 17 K for less than 1 hour to prompt the release of H_2_ from the gold-plated copper walls of the microscope cryostat (*40*).

### Computational details

DFT calculations were performed using the VASP code (*41*). The Perdew-Burke-Ernzerhof (*42*) form of generalized gradient approximation was used as an exchange and correlation functional. Core electrons were treated following the PAW method (*43*). Two supercell structures were used, both representing epitaxially extended Co bilayers on a (111) oriented Cu substrate. The first one is a 2 × 2 − (111) supercell including two layers of Co (4 Co atoms per layers) and nine layers of underlying Cu (4 Cu atoms per layer). It enables to achieve surface hydrogen coverages between 0.25 and 1 ML by the addition of 1 to 4 hydrogen atoms in hollow sites. Possible insertion in the most stable inner octahedral sites was also considered with four octahedral sites available at the CoCo and CoCu interfaces, i.e., 8 in total or 12 including the hollow surface adsorption sites. The second geometry used is the simple 1 × 1 −(111) supercell including two layers of Co (1 Co atom per layer) and nine layers of underlying Cu (1 Cu atom per layer). In this case, the addition of one hydrogen atom at the surface or at the CoCo and CoCu interfaces in interstitial position, corresponds to full hydrogen layers. A vacuum layer of 20 Å was always added in the direction perpendicular; hence, periodic images and slab asymmetry can be safely neglected (*44*). In any case, an energy cutoff of 500 eV is used. Then, for the 2 × 2 cell, a 12 × 12 × 1 *k*-point sampling was applied, while a larger 30 × 30 × 1 *k*-point sampling was used for the smaller 1 × 1 cell. Considering pristine Co and full adsorbed or inserted H layers, we checked that using one cell geometry or the other, with its associated *k*-point set, led to the same magnetocrystalline anisotropy energies with an error of less than 20 μeV per atom.

Calculations are performed into two main steps following the magnetic force theorem (*45*), one involving spin-polarized calculations in the collinear scheme and the other including spin-orbit coupling (SOC). In the collinear case, the supercell is relaxed along the *z* direction while the lateral lattice constant is fixed to the one of Cu (*a* = 3.635 Å, obtained after optimization of the copper substrate alone) according to an epitaxial growth. The positions of all atoms except for those in the two bottom layers were relaxed (along z direction) until all forces were smaller than 0.01 eV/Å and the total energy converged within an accuracy of 1 × 10^−7^ eV. At this stage, the output charge densities are used to deduce the spin densities, whose maps are plotted with the VESTA software (*46*), and STM images are simulated following the Tersoff-Hamann theory (*47*) using the STMpw code (*48*). The same charge densities are also used in order to perform calculations including the spin-orbit interaction as implemented in VASP (*45*). The MCA was determined by rotating the spins according to different crystallographic directions. In our case, the spin-orbit coupling was taken into account non–self-consistently for the spin orientations corresponding to in-plane and out-of-plane magnetizations, respectively. Therefore, the total calculated MCA is the energy difference between a system with all spins out of plane and a system with all spin in plane. Then, the site-resolved MCA is obtained by the decomposition of the total MCA through a projection of the corresponding rotated wave functions on site orbitals. More precisely, we get a SOC energy $\left(E_{\text{soc}}\right)$ on each ion, which represents the accumulated energy contribution inside the augmentation sphere that is centered at each ion position. To determine the total magnetic anisotropy, we add to the MCA the so-called shape anisotropy, which results from magnetostatic dipole-dipole interactions and therefore depends on the geometry of the system under study. In the case of the infinite bilayers of the present work, this contribution $E_{\mathrm{dd}}$ was evaluated numerically using the following summation up to an in-plane cutoff radius of 150 Å (*49*)

$$E_{\mathrm{dd}}=\frac{\mu_{0}}{8\pi }\sum_{i\neq j}\frac{1}{{|r_{\mathrm{ij}}|}^{3}}\left[m_{i}\cdot m_{j}-3\frac{\left(r_{ij}\cdot m_{i}\right)\cdot \left(r_{ij}\cdot m_{j}\right)}{{|r_{\mathrm{ij}}|}^{2}}\right]$$ (2)

This cutoff radius is sufficiently large to ensure well-converged shape anisotropy energies while accurately representing the properties of the infinite layer. The total MAE, between out-of-plane (⊥) and in-plane magnetization (∥), is therefore given by the difference of energies

$$\begin{array}{c} \text{MAE}=\text{MCA}+\Delta E_{\mathrm{dd}}\\ =\left(E_{\text{tot},⊥}^{\text{DFT}}-E_{\text{tot},∥}^{\text{DFT}}\right)+\left(E_{\mathrm{dd},⊥}-E_{\mathrm{dd},∥}\right) \end{array}$$ (3)

Using this latter equation leads to positive (negative) values of MAE for in-plane (out-of-plane) orientation.
