# Supplementary material for: Molecular spin-probe sensing of H-mediated changes in Co nanomagnets
Source: Sci Adv. 2025 Feb 12;11(7):eads1456. doi: 10.1126/sciadv.ads1456 (PMC11817921; doi:10.1126/sciadv.ads1456)
Supplement: Supplementary file 1 — Supplementary Text Figs. S1 to S8 Table S1 [file sciadv.ads1456_sm.pdf]

Science Advances
AAAS

# Supplementary Materials for

## Molecular spin-probe sensing of H-mediated changes in Co nanomagnets

Alex Fétida *et al.*

Corresponding author: Laurent Limot, limot@ipcms.unistra.fr

*Sci. Adv.* **11**, eads1456 (2025)
DOI: 10.1126/sciadv.ads1456

**This PDF file includes:**

Supplementary Text
Figs. S1 to S8
Table S1

# Supplementary Text

## Low- and high-bias imaging

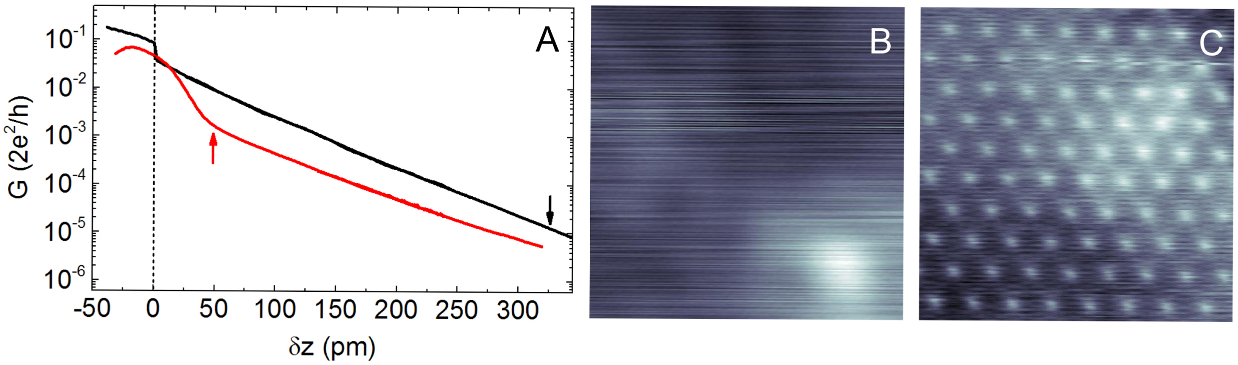


**Figure S1**: **Imaging with a nickelocene tip: Tip-surface distance dependence.** Conductance versus tip-sample distance acquired with an Nc-tip above a Co bilayer. The traces are obtained using two different feedback-loop opening parameters: 50 pA and 50 mV (solid black line) and 100 pA and 1 mV (solid red line). The opening positions are marked by arrows. The dashed line corresponds to the contact point between the Nc-tip and the Co surface. (**B**) Image of a Co bilayer with an Nc-tip ($2 \times 2$ nm$^2$, 50 mV, 50 pA). (**C**) Image of a Co bilayer with the same Nc-tip as panel (B) but acquired with different tunneling parameters ($2 \times 2$ nm$^2$, 1 mV, 100 pA).

To investigate hydrogen exposure to cobalt, we employ two distinct operating modes of the molecular tip, as illustrated in fig. S1A, which shows two conductance ($G$) versus tip displacement ($\delta z$) traces obtained above a pristine Co bilayer island. The first trace (solid black line) is acquired by opening the feedback loop at 50 pA and 50 mV. The trace is exponential, with a sharp change in slope marking the contact with the Co surface *(50)*. From this trace, we deduce that the Nc-tip is approximately 325 pm from contact when the feedback loop is opened with these parameters (black arrow in fig. S1A). The exchange interaction is absent in this case, resulting in a featureless image of the Co island (fig. S1B). Thus, the Nc-tip functions as a standard STM tip. The second trace in fig. S1A (solid red line) is obtained using the same Nc-tip by opening the feedback loop at 100 pA and 1 mV. This trace exhibits a faster-than-exponential behavior before reaching contact. From this trace, we deduce that the Nc-tip is 50 pm away from contact when the feedback loop is opened with this tunnel current and bias (red arrow in fig. S1A). In this case, the exchange interaction is active, and

when scanning the Co surface, a magnetic corrugation is observed (fig. S1C). In the experiment, we use these two sets of tunneling parameters: 1 mV and 100 pA for an active exchange interaction (low-bias mode), and 50 mV and 50 pA for the absence of exchange interaction (high-bias mode). This approach allows us to visualize the electronic and magnetic properties independently.

**Computed images and LDOS of H-covered cobalt**

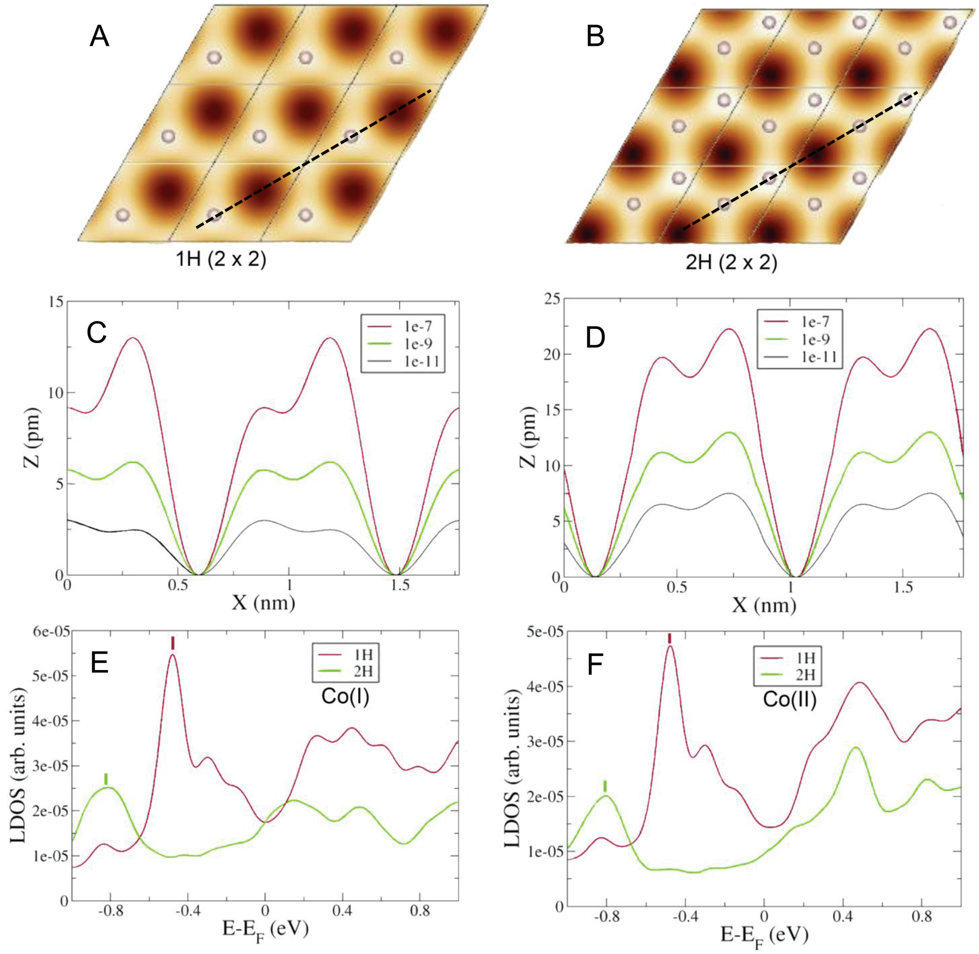


**Figure S2**: **Computed images and LDOS of H-covered cobalt.** (**A**)-(**B**) Computed constant-current images of the 1H-($2 \times 2$) and 2H-($2 \times 2$) superstructures on a cobalt bilayer on Cu(111), respectively. The images were computed using the Tersoff-Hamann approximation with 0.1 V. Circles represent hydrogen atoms. Panels (**C**) and (**D**) show the height profiles acquired along the dashed lines indicated in (A) and (B), respectively for various tunneling currents (in arbitrary units). The superstructures exhibit similar corrugations. Panels (**E**) and (**F**) present the computed LDOS at 300 pm above Co(I) and Co(II), respectively. The solid red lines represent the LDOS for cobalt in the 1H-($2 \times 2$) superstructure, while the solid green lines represent the LDOS for cobalt in the 2H-($2 \times 2$) structure. The vertical tics indicate the position of the dominant $d$-peak observed in tunneling spectroscopy at negative bias.

**Conductance versus tip displacement above cobalt**

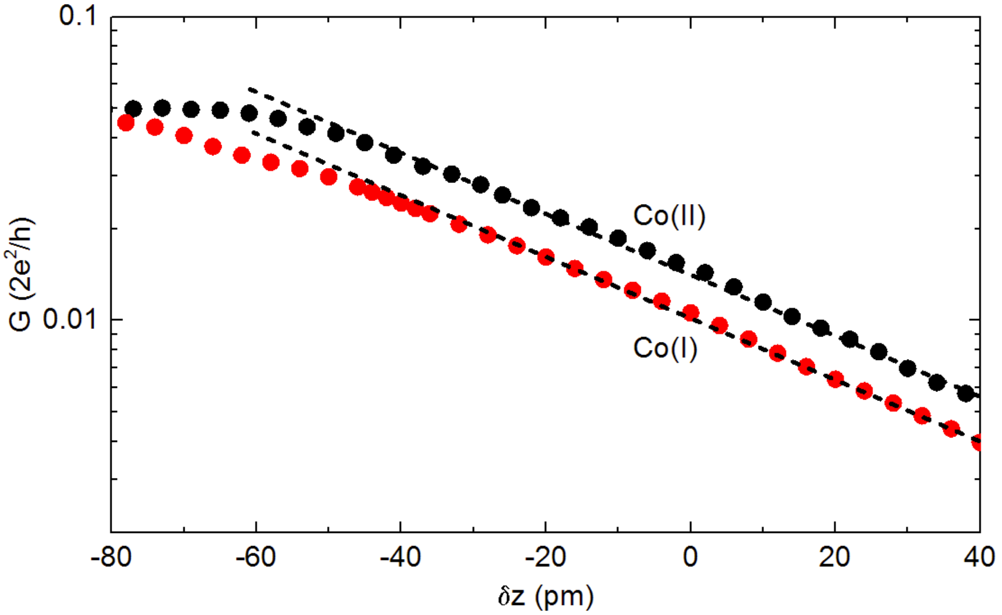


**Figure S3**: **Conductance versus tip-Co distance.** Conductance versus tip displacement traces acquired above Co(I) (red circles) and Co(II) (black circles) using an Nc-tip. The feedback loop was opened above Co(I) at 11 mV and 9 nA.

Prior to investigating the magnetic properties, we focus on calibrating the tip-sample distance. Figure S3 presents typical traces acquired above Co(I) (red circles) and Co(II) (black circles). The traces above Co(I) and Co(II) differ from those acquired above the pristine cobalt island (fig. S1A) exhibiting a change of the exponential decay length around $G = 0.03\, G_0$ ($G_0 = 2e^2/h$), which is more pronounced for Co(II) than Co(I). The conductance remains well below $G_0$ and no clear inflection is observed. This behavior resembles the conductance observed when approaching an organic molecule *(26)* or a CoH complex *(8)* with a hydrogen-functionalized tip. This results from tip-sample repulsion at small distances, which causes the vacuum gap to shorten more slowly than expected as the piezoelectric tube extends. Since the contact point cannot be clearly defined, we express the distance in terms of a tip displacement ($\delta z$). The zero displacement $\delta z = 0$ is set to a conductance of $G = 0.01\, G_0$ above a Co(I) atom.

## Magnetism of Co(I) and Co(II)

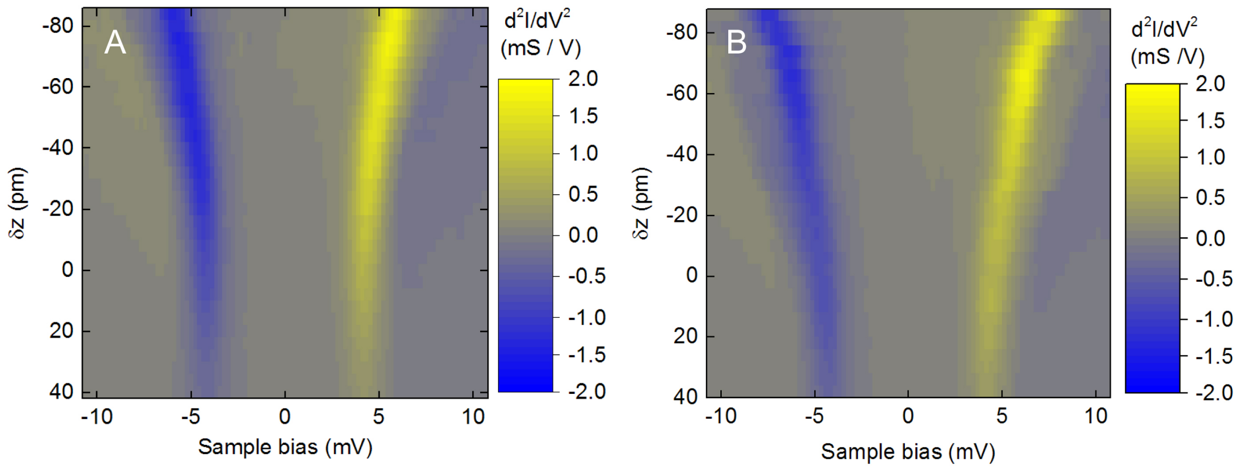


**Figure S4**: **2D intensity plots above the Co sites.** Panels (**A**) and (**B**) present typical 2D intensity plots acquired above Co(I) and Co(II) for different tip displacements, respectively.

**Spin polarization of Co(I) and Co(II)**

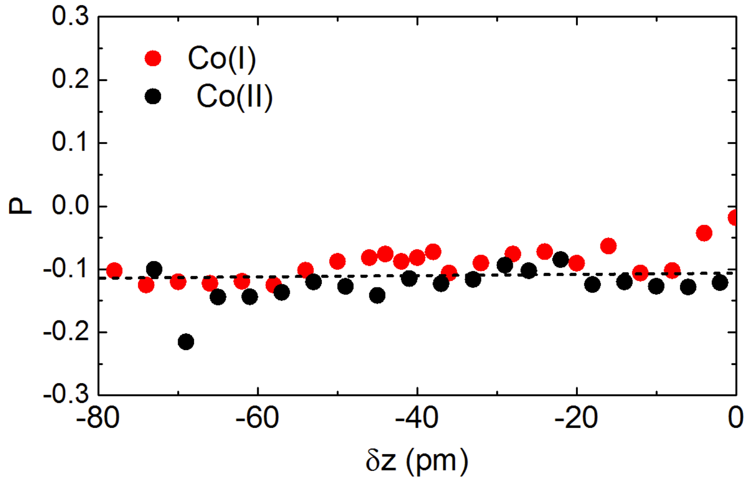


**Figure S5**: **Spin polarization versus tip-Co distance.** Spin polarization versus $\delta z$ from the $d^2I/dV^2$ spectra of Co(I) and Co(II). The dashed line is a guide to the eye. The zero displacement $\delta z = 0$ is set to a conductance of $G = 0.01\,G_0$ above a Co(I) atom (see fig. S3). The different spin polarization found compared to the pristine surface where $P = 0$ *(30,31)* indicates that the DOS of both types of Co atoms has changed, consistent with our LDOS calculations of fig. S2.

For $\delta z < -40$ pm (Fig. 3A), the exchange energy becomes less sensitive to further displacement, likely due to Nc-H repulsion. Since surfaces with in-plane magnetization show varying spin polarization with Nc tilt angle *(31)*, the constant spin polarization of $P = -0.12$ for both Co(I) and Co(II) (fig. S5) suggests that the Nc tilt angle remains stable as the tip approaches the Co atoms. We therefore infer that Nc is compressed toward the metal tip, rather than tilting.

**Exchange maps of H-covered Co islands**

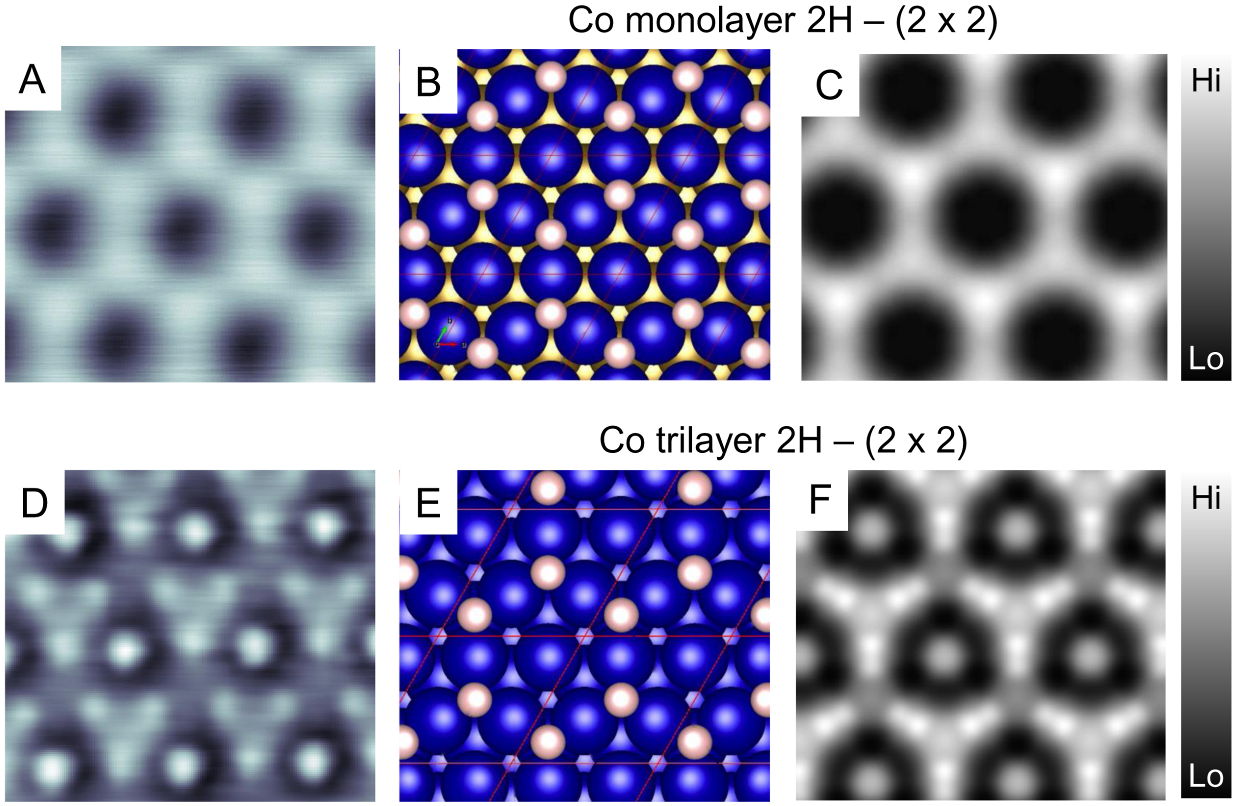


**Figure S6**: **Exchange and computed spin-density maps for varying island thicknesses.** (**A**) Low-bias image ($1.2 \times 1.1$ nm$^2$) acquired with a Nc-tip positioned above a Co monolayer on Cu(111) covered with hydrogen. (**B**) Model structure of a Co monolayer with 2H-($2 \times 2$) superstructure (Co: blue, H: white, Cu: yellow) and (**C**) Corresponding DFT-computed spin density map 200 pm above the H atoms. Panels (**D**) to (**F**) present same results as for panels (A) to (C) but acquired on a Co trilayer on Cu(111) covered with hydrogen. The spin density (F) is also computed 200 pm above the H atoms.

**Orbital-resolved analysis of the MCA for a Co bilayer**

The magnetocrystalline anisotropy Energy (MCA), stems from the spin-orbit coupling (SOC). The SOC interaction is described by the Hamiltonian $H_{SOC} = \xi(\hat{L} \cdot \hat{S})$, where $\xi$ is the SOC constant, $\hat{L}$ is the orbital angular momentum operator, and $\hat{S}$ is the spin operator. This term couples the electron spin state with its orbital state, leading to energy shifts that depend on the relative orientation of the spin and orbital moments. It dictates the preferred alignment direction of magnetic moments within the material arising from the crystalline structure and symmetry. The different MCA for surface and first-layer Co atoms can be attributed to their hybridization with H atoms. For instance, it has been shown that hydrogen absorption in Pd/Co/Pd thin films changes the relative importance of different *d* orbitals in their contribution to MCA *(19)*. To elucidate the relation between MCA and *d* orbitals, we use our DFT calculations including SOC to compute the matrix elements $\langle\psi_i|\hat{L} \cdot \hat{S}|\psi_j\rangle$, where $|\psi\rangle$ corresponds to a $3d$ state of Co. These matrix elements allow us to estimate the contribution to MCA from each pair $(\psi_i, \psi_j)$ of $d$ atomic-like orbitals.

The orbital-pair contributions to the MCA are represented as bar diagrams in fig. S7, where we compare a pristine and an H-covered Co bilayer. In particular, we consider a Co bilayer with its surface fully loaded with hydrogen (1 ML coverage) to highlight the impact of Co-H hybridization on the MCA. As shown in fig. S7, only three pairs of orbitals have a sizable contribution to the MCA: $(yz, z^2)$, $(yz, xz)$, and $(xy, x^2 - y^2)$. For the pristine Co bilayer (fig. S7A), similar observations can be made for both the surface Co layer (top panel) and the first Co layer (bottom panel). The orbital pair $(xy, x^2 - y^2)$, which includes orbitals parallel to the surface, gives the strongest contribution to MCA and favors an out-of-plane orientation of the magnetization. The $(yz, xz)$ orbital pair contributes less to MCA while also favoring an out-of-plane orientation. The $(yz, z^2)$ orbital pair, which includes orbitals perpendicular to the surface, favors an in-plane orientation. Overall, the MCA of both the surface and the first layer is negative (green line in fig. S7A). Upon adsorption of 1 ML of hydrogen and its hybridization with the Co surface atoms, drastic changes are observed (fig. S7B). The out-of-plane MCA related to $(xy, x^2 - y^2)$ is suppressed in the Co surface layer (top panel), favoring in-plane magnetization for the surface. Consequently, the MCA of the surface becomes positive (green line in fig. S7B). This change is partly compensated in the Co first layer by the increased contribution to MCA of the $(yz, xz)$ orbital pair (bottom panel), which favors

out-of-plane magnetization. Overall, hydrogen hybridization with Co drives complex changes in the MCA of the Co bilayer but is insufficient alone to cause a rotation of the magnetization from out-of-plane to in-plane. Only when considering the contribution of shape anisotropy can a reversal of the orientation be achieved.

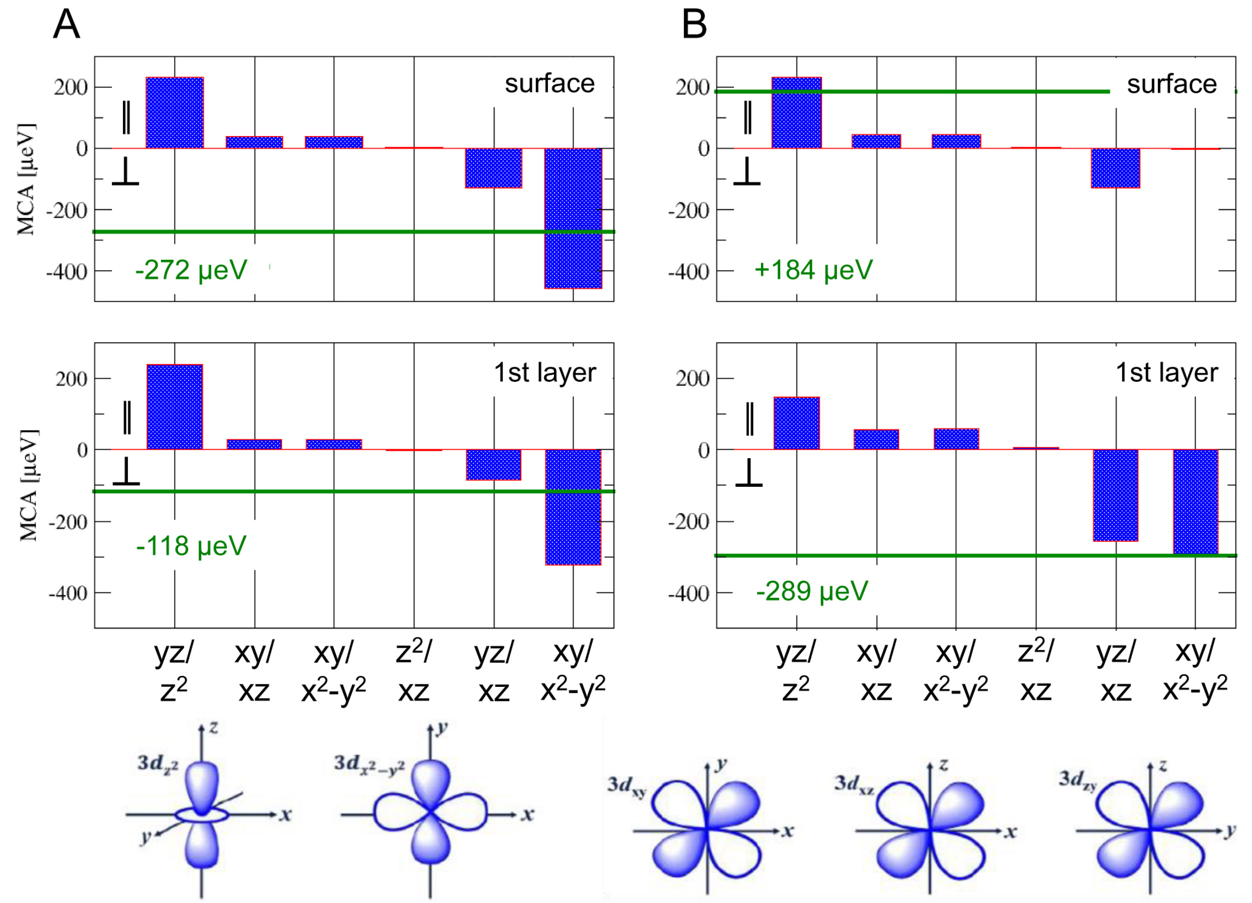


**Figure S7**: **Orbital-resolved analysis of the MCA for Co in the presence of hydrogen.** (**A**) Pristine Co bilayer, (**B**) A Co bilayer with a 1 ML hydrogen-covered surface. The top and bottom panels correspond to the Co surface and to the Co of the first layer, respectively. The green line corresponds to the total MCA per layer. The computation uses a Co supercell-$1 \times 1$ and $36 \times 36 \times 1$ $k$-points to ensure the best possible precision.

## Hydrogen insertion in the Co bilayer

Table S1 presents DFT-computed values for the magnetic anisotropy energy (MAE, in eV/cell) and total energy ($E_{tot}$, in eV) for various configurations in which hydrogen (H) is inserted into the Co bilayer. The top Co layer, which is exposed to vacuum, exhibits a 2H-$(2 \times 2)$ structure, resulting in a H coverage of 0.5 ML [panel (A)]. In panels (B)–(D), we explore the effects of hydrogen insertion within the Co bilayer. Two distinct interfaces are considered: i) Between the top and bottom Co layers of the island, referred to as the Co/Co interface, where the unit cell consists of a $2 \times 2$ Co structure. ii) Between the bottom Co layer and the Cu substrate, referred to as the Co/Cu interface, where the unit cell is a $2 \times 2$ Cu structure. In panel (B), one hydrogen atom is inserted at the Co/Co interface. In panels (C) and (D), one and two hydrogen atoms, respectively, are inserted at the Co/Cu interface. Hydrogen atoms can occupy various octahedral sites within the Co bilayer, resulting in multiple configurations for each hydrogen coverage. For each coverage, we retain only the configurations with the lowest energy.

Adding hydrogen at the Co/Co interface (tab. S1B) consistently results in a significant increase in out-of-plane magnetization (MAE $< 0$) compared to the initial configuration where hydrogen is only present on the island surface (tab. S1A). This outcome is not consistent with experimental observations. Conversely, inserting hydrogen at the Co/Cu interface causes a marked increase in MAE towards positive values. The bold values in tab. S1C and S1D indicate configurations with net in-plane magnetization (MAE $> 0$) and have been used in Fig. 4A of the main text to illustrate the enhancement of in-plane magnetization through hydrogen insertion at the Co/Cu interface. Considering all configurations for each coverage, the configuration-averaged MAE is +0.004 eV/cell for 0.75 ML and +0.136 eV/cell for 1 ML. These results confirm the overall trend: hydrogen insertion at the Co/Cu interface leads to a substantial increase in MAE, promoting an evolution towards in-plane magnetization.

| (**A**) H-coverage of 0.5 ML | | |
|---|---|---|
| 2H-($2 \times 2$) at surface, no H inserted in the bilayer | | |
| Configuration | MAE (eV/cell) | $E_{tot}$ (eV) |
| 1 | -0.229 | -192.952 |

| (**B**) H-coverage of 0.75 ML | | |
|---|---|---|
| 2H-($2 \times 2$) at surface (0.5 ML), 1 H at Co/Co interface (0.25 ML) | | |
| Configuration | MAE (eV/cell) | $E_{tot}$ (eV) |
| 1 | -0.436 | -196.063 |
| 2 | -0.489 | -196.140 |
| 3 | -0.429 | -196.141 |
| 4 | -0.502 | -196.138 |

| (**C**) H-coverage of 0.75 ML | | |
|---|---|---|
| 2H-($2 \times 2$) at surface (0.5 ML), 1 H at Co/Cu interface (0.25 ML) | | |
| Configuration | MAE (eV/cell) | $E_{tot}$ (eV) |
| 1 | **+0.192** | -196.132 |
| 2 | -0.055 | -196.142 |
| 3 | -0.064 | -196.145 |
| 4 | -0.059 | -196.142 |

| (**D**) H-coverage of 1 ML | | |
|---|---|---|
| 2H-($2 \times 2$) at surface (0.5 ML), 2 H at Co/Cu interface (0.5 ML) | | |
| Configuration | MAE (eV/cell) | $E_{tot}$ (eV) |
| 1 | **+0.344** | -199.360 |
| 2 | -0.073 | -199.376 |

**Table S1**: **DFT results for hydrogen inserted at different sites in the Co bilayer.** The first and second columns show the magnetic anisotropy energy (MAE, eV/cell) and total energy ($E_{tot}$, eV), respectively. Panel (**A**) is the reference system with 2H-($2 \times 2$) surface coverage and no inserted H. Panels (**B**)–(**D**) show configurations with additional H: (B) 1 H at the Co/Co interface, (C) 1 H at the Co/Cu interface, and (D) 2 H at the Co/Cu interface.

**Subsurface spin-sensitivity: Pristine Co bilayer**

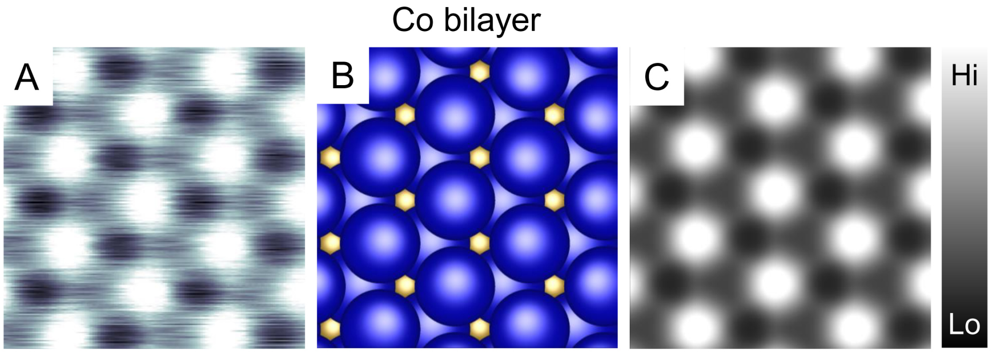


**Figure S8**: **Low-bias image and computed spin-density map of a pristine Co island.** (**A**) Low-bias image (0.5 mV, 600 pA, $0.8 \times 0.8$ nm$^2$) acquired with a Nc-tip positioned above a pristine Co bilayer on Cu(111). (**B**) Model structure of a Co bilayer (Co: blue, Cu: yellow), and (**C**) Corresponding DFT-computed spin density map 300 pm above the Co atoms.

The Nc-tip demonstrates spin sensitivity to Co atoms on the bottom layer of the island, *i.e.* to the Co atoms at the interface with the copper substrate *(30)*. A typical image showing this "subsurface" sensitivity is presented in fig. S8A and reveals distinct exchange energies at the *hcp* and *fcc* sites of the Co island. The strongest signal is assigned to the *fcc* site and the weakest to the *hcp* site (fig. S8B) based on the DFT-computed spin density map (fig. S8C).
